# Cardiovascular risk factors in patients with premature myocardial infarction and in their first-degree relatives

**DOI:** 10.1186/1758-5996-7-S1-A162

**Published:** 2015-11-11

**Authors:** Maria Helane da Costa Gurgel Castelo, Renan Magalhães Montenegro, Clarisse Mourão Melo Ponte, Tamara Cristina S Sousa, Paulo Goberlanio B Silva, Lucia de Sousa Belém, Alexandre C Pereira, Raul Dias Santos Filho

**Affiliations:** 1 Faculdade de Medicina da universidade Federal do Ceará, Fortaleza, Brazil

## Background

Acute myocardial infarction (AMI) is unusual in people before age 45 and is related to premature family history of cardiovascular disease.

## Objective

Describe socio-demographic and cardiovascular risk factors of subjects with AMI<45 yrs. old and their first-degree relatives. Evaluate association of clinical and laboratory parameters with angiographic extension of coronary artery disease (CAD).

## Materials and methods

Cross-sectional study conducted in a tertiary hospital (November/2010 – January/2015). We included 103 index cases and 166 first-degree relatives without suspicion of familial hypercholesterolemia, compared with 111 asymptomatic individuals without family history of CAD matched for sex and age. Clinical and laboratory parameters were evaluated. Associations were tested by statistical analysis.

## Results

AMI cases had higher prevalence of smoking (57.3% vs. 28.6%, p<0.001), type 2 diabetes mellitus -DM2 (43.4 vs. 19.5%, p<0.001), and hypertension (42.7 vs. 19%, p<0.001), when compared to relatives. When compared to controls, cases showed higher triglycerides (192±75mg/dL vs. 140±74mg/dL, p<0.001), and metabolic syndrome –MS (82.2% vs. 36%, p<0.001), and lower HDL-c (36±12mg/dL vs. 48±14mg/dL, p<0.001). Multivessel disease was found in 50.5% of cases. It was independently associated with hypertension (p=0.030), and DM2 (p=0.028) after multivariate analysis. In comparison to controls, relatives had greater prevalence of smoking (29.5% vs. 6.3%, p<0.001), DM2 (19.9% vs. 1.8%, p <0.001), pre-diabetes (40.4% vs. 27%, p<0.024) and MS (64.7% vs. 36%, p<0.001), lower HDL-c (39±10mg/dL vs. 48±14mg/dL, p<0.001), higher triglycerides (179±71mg/dL vs. 140±74mg/dL, p=0.002), higher LDL-c (122±37mg/dL vs. 113±36mg/dL, p=0.031), non-HDL cholesterol (157±43 vs. 141±41mg/dL, p=0.004), and higher prevalence of high/intermediate calculated coronary heart disease risk according to Framingham risk score (82.7% vs. 2.6%, p<0.001). There was no difference in BMI (p=0.051). TSH levels even within the reference value method were higher in AMI patients (2.6±1.6mUI/mL, p<0.001) and relatives (2.4±1.6mUI/mL, p=0.002) in comparison with controls 1.9±1.0mUI/mL).

## Conclusion

High prevalence of risk factors mainly MS, atherogenic dyslipidemia, DM2, hypertension and smoking were encountered in cases and first-degree relatives of individuals with AMI <45 yrs. Hypertension and DM2 were associated with greater angiographic extent of coronary artery disease.

